# Unequal Progress in Early-Onset Bladder Cancer Control: Global Trends, Socioeconomic Disparities, and Policy Efficiency from 1990 to 2021

**DOI:** 10.3390/healthcare14020193

**Published:** 2026-01-12

**Authors:** Zhuofan Nan, Weiguang Zhao, Shengzhou Li, Chaoyan Yue, Xiangqian Cao, Chenkai Yang, Yilin Yan, Fenyong Sun, Bing Shen

**Affiliations:** 1 Department of Urology, Shanghai General Hospital, Shanghai Jiao Tong University School of Medicine, Shanghai 200080, China; 2 Department of Urology, Shanghai Tenth People’s Hospital, School of Medicine, Tongji University, Shanghai 200072, China; 3 Huadong Hospital, Shanghai Medical College, Fudan University, Shanghai 200040, China; 4 Obstetrics and Gynecology Hospital, Fudan University, Shanghai 200011, China; 5 Department of Clinical Laboratory, Shanghai Tenth People’s Hospital, School of Medicine, Tongji University, Shanghai 200072, China

**Keywords:** bladder cancer, early-onset cancer, global burden of disease, SDI, cross-sectional study

## Abstract

**Background**: This study investigates the global burden of early-onset bladder cancer (EOBC) from 1990 to 2021, highlighting regional disparities and the growing role of metabolic risk factors. Early-onset bladder cancer (EOBC), diagnosed before age 50, is an emerging global health concern. While less common than kidney cancer, EOBC contributes substantially to mortality and disability-adjusted life years (DALYs), with marked sex disparities. Its global epidemiology remains unassessed systematically. **Methods**: Using GBD 1990–2021 data, we analyzed EOBC incidence, prevalence, mortality, and DALYs across 204 countries in individuals aged 15–49. Trends were examined via segmented regression, EAPC, and Bayesian age-period-cohort modeling. Inequality was quantified using SII and CI. Decomposition and SDI-efficiency frontier analyses were introduced. **Results**: From 1990 to 2021, EOBC incidence rose 62.2%, prevalence 73.1%, deaths 15.3%, and DALYs 15.8%. Middle-SDI regions bore the highest burden. Aging drove trends in high-SDI areas and population growth in low-SDI regions. Over 25% of high-SDI countries underperformed in incidence/prevalence control. Smoking remained the leading risk factor, with rising hyperglycemia burdens in high-income areas. Males carried over twice the female burden, peaking at age 45–49. **Conclusions**: EOBC shows sustained global growth with middle-aged concentration and significant regional disparities. Structural inefficiencies highlight the need for enhanced screening, early warning, and tailored resource allocation.

## 1. Introduction

Bladder cancer ranks as the ninth most common malignancy worldwide, yet its early-onset form (EOBC, diagnosed at ages 15–49) remains an under-recognized contributor to the global cancer burden [1]. Recent epidemiological evidence from the Global Burden of Disease (GBD) 2021 dataset indicates that early-onset cancers overall have risen sharply over the past three decades, with incidence increasing by 79.1% and mortality by 27.7% between 1990 and 2021 [2]. EOBC, while less prevalent than early-onset kidney cancer, carries higher mortality than early-onset prostate cancer and causes disability-adjusted life years (DALYs) comparable to kidney cancer. The disease demonstrates stark gender disparities (male-to-female incidence ratio approaching 3:1) [3], and its muscle-invasive forms often require prolonged and costly treatments, including radiotherapy, chemotherapy, and radical cystectomy. These interventions impose substantial, long-term health and economic burdens on patients in their most productive years, with impacts that far exceed earlier estimates [4]. Despite these trends, EOBC remains marginalized in research funding, clinical guidelines, and public health policy [5].

Recent studies, such as Su et al. [6], systematically reviewed global early-onset genitourinary cancers using GBD data, highlighting EOBCs significant burden in terms of mortality and DALYs. However, it did not perform a refined decomposition analysis across population growth, aging, and epidemiological changes. Crucially, the absence of a frontier shift model limited deeper insights into the underlying epidemiological drivers of EOBC [7]. Other EOBC-focused studies suffer from regional/population constraints or methodological shortcomings, precluding comprehensive assessments [2,3,7,8,9,10,11,12].

Based on GBD 1990–2021 data, this study provides a comprehensive epidemiological assessment of EOBC burden across 204 countries and territories. Age- and sociodemographic index (SDI)-stratified analyses were performed to quantify incidence, mortality, prevalence, and disability-adjusted life years (DALYs). Inequality metrics—including the slope index of inequality (SII) and concentration index (CI)—and temporal trend models (AAPC/APC) were incorporated, and Bayesian age-period-cohort modeling projected burdens to 2050.

## 2. Materials and Methods

### 2.1. Data Collection and Sources

Early-onset bladder cancer (EOBC) was defined as cases occurring among individuals aged 15–49 years, which encompasses both adolescents and young adults (AYA) [6,7,13]. The age range of 15–49 years was selected to capture the full spectrum of early-onset bladder cancer cases across young adulthood, as it is consistent with global health initiatives and existing cancer registries that use this range [8,12].

Data were obtained from the Global Burden of Disease (GBD) 2021 study, which provides estimates for 369 diseases, injuries, and 88 risk factors across 204 countries and territories from 1990 to 2021. Bladder cancer was defined according to the International Classification of Diseases (ICD) codes—ICD-10: C67 and ICD-9: 188—as used in the GBD cause list. The coding framework has remained stable across the study period, with cross-mapping between ICD versions ensuring consistency.

We extracted EOBC-specific estimates for mortality, prevalence, incidence, and disability-adjusted life years (DALYs) from the Global Health Data Exchange (GHDx). The Socio-demographic Index (SDI) was used to assess socioeconomic influences on EOBC burden.

### 2.2. Trend Analysis

Temporal trends in EOBC incidence, prevalence, mortality, and DALYs from 1990 to 2021 were evaluated using the Joinpoint Regression Program (version 4.9.0.0; National Cancer Institute, Bethesda, MD, USA), which fits the simplest model permitted by the data to identify statistically significant changes in trends. Annual Percentage Change (APC) and Average Annual Percentage Change (AAPC) were calculated with 95% confidence intervals (CIs).

### 2.3. Decomposition Analysis

We further evaluated EOBC distribution across demographic subgroups, stratifying data into seven age categories for both sexes. To identify the main drivers of temporal changes, decomposition analysis partitioned these changes into population growth, population aging, and epidemiologic variations [14,15].

### 2.4. Inequality Metrics

Inequality was assessed using two indices: the Slope Index of Inequality (SII) and the Concentration Index (CI). These measures help capture regional disparities in EOBC burden relative to socio-economic development.

### 2.5. Frontier Analysis

The frontier shift model was used to determine the theoretical minimum age-standardized rates (ASRs) of EOBC, with the efficiency gap serving as an indicator of healthcare system performance. The SDI–ASR efficiency frontier was constructed using a boundary-fitting method linking the minimum ASRs observed across SDI values. For each SDI level, the frontier denotes the lowest theoretically attainable burden, and the deviation of observed ASR from this frontier quantifies the efficiency gap in disease control performance. This approach helps identify regions that have deviated the most from optimal disease burden levels, providing insights into healthcare inequalities and efficiency.

### 2.6. Risk Analysis

Risk factors contributing to the EOBC burden were identified using data from the Global Burden of Disease (GBD) 2021 study. Among all quantified risk factors, smoking and high fasting plasma glucose (FPG) were included as the two leading modifiable contributors to EOBC-related disability-adjusted life years (DALYs) and mortality.

### 2.7. Bayesian Age–Period–Cohort (BAPC) Model

Future incidence and prevalence of EOBC to 2050 were projected using a Bayesian age–period–cohort (BAPC) model implemented through integrated nested Laplace approximation (INLA) in R. The model assumes a Poisson likelihood with a log link function, and random walk (RW2) priors were specified for age, period, and cohort effects to ensure smoothness. Over-dispersion was accounted for by including a Gaussian random effect. Uncertainty intervals (95% UIs) for the projections were derived from the posterior distributions obtained through INLA-based Bayesian inference [16].

### 2.8. Software and Statistical Tools

All analyses were performed using R software (version 4.4.1) with the ggplot2 and sf packages [17]. Results are presented as means with 95% uncertainty intervals (UIs) or 95% CIs. A *p*-value < 0.05 was considered statistically significant.

## 3. Results

### 3.1. The Global Trend of Early-Onset Bladder Cancer Burden from 1990 to 2021

From 1990 to 2021, the global incidence of early-onset bladder cancer (EOBC) in individuals aged 15–49 years increased significantly. The number of new cases rose by 62.2%, from 19,146.94 (95% UI: 16,119.05, 20,578.77) to 31,053.76 (95% UI: 28,341.92, 34,322.02). The age-standardized rate (ASR) of incidence increased by 11.33%, from 0.71 (95% UI: 0.59, 0.76) to 0.79 (95% UI: 0.72, 0.87).

The number of EOBC-related deaths increased by 15.3%, from 5486.52 (95% UI: 4460.69, 5976.30) to 6327.78 (95% UI: 5732.75, 6995.74). Meanwhile, the death ASR declined by 20.83%, from 0.20 (95% UI: 0.16, 0.22) to 0.16 (95% UI: 0.15, 0.18).

The estimated annual percentage changes (EAPCs) for the incidence ASR and death ASR were 0.09 (95% CI: −0.06, 0.23) and −0.77 (95% CI: −0.89, −0.65), respectively (Table 1 and Table 2).

Prevalence increased by 73.1%, while DALYs rose by 15.8%. The prevalence of ASR increased from 10.70 (95% UI: 10.37, 10.99) to 12.64 (95% UI: 12.18, 13.18), whereas the DALYs of ASR decreased from 11.74 (95% UI: 11.17, 12.18) to 9.59 (95% UI: 9.04, 10.24).

The EAPCs for prevalence and DALYs ASRs were 0.31 (95% CI: 0.16, 0.47) and −1.05 (95% CI: −1.18, 0.93), respectively (Tables S1 and S2).

### 3.2. Trends of Regions Defined by Socio-Demographic Index (SDI)

Globally, in 2021, the burden of EOBC demonstrated marked geographic heterogeneity. Overall, the trend across the socio-demographic index (SDI) spectrum showed that incidence, mortality, prevalence, and DALYs first increased and then decreased as SDI decreased. More specifically, the highest burden was observed in middle-SDI regions, which reported 8886.95 (95% UI: 7730.48, 10,264.97) new cases and 1952.80 (95% UI: 1721.06, 2255.05) deaths. Corresponding prevalence and DALYs were 67,820.30 (95% UI: 58,855.93, 78,724.27) and 99,594.74 (95% UI: 87,511.54, 114,203.99), respectively. Geographically, incidence, mortality, prevalence, and DALYs were concentrated in East/South/Southeast Asia, Western Europe, high-income North America, North Africa, and the Middle East, whereas Andean Latin America, Oceania, and Australasia showed the most favorable profiles.

**Figure 1:**
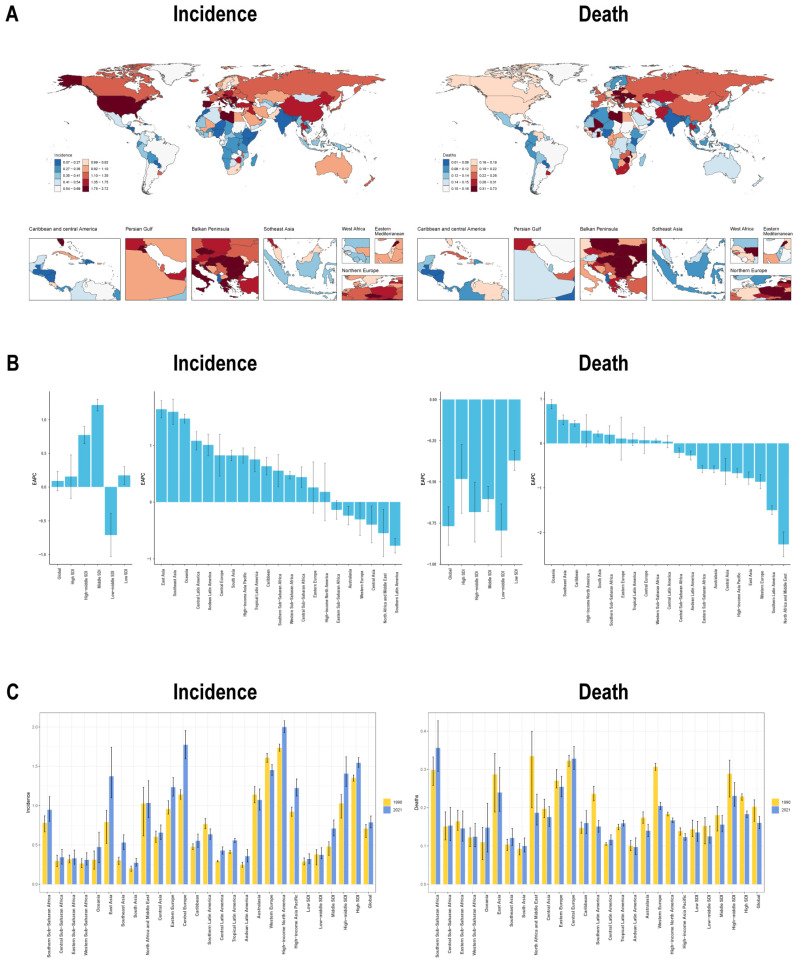
The global burden distribution of early-onset bladder cancer through three panels: (**A**) Heatmaps showing EAPCs for incidence (**left**) and mortality (**right**) worldwide; (**B**) Bar charts visualizing the same EAPC data; (**C**) Comparative bar plots displaying ASRs for incidence (**left**) and mortality (**right**) across 21 regions in 1990 versus 2021.

Notably, the EAPCs of ASRs for incidence and prevalence were highest in middle-SDI regions—1.22 (95% CI: 1.13, 1.30) and 1.65 (95% CI: 1.58, 1.72), respectively—and lowest in low-middle SDI regions at −0.71 (95% CI: −1.03, −0.39) and −0.60 (95% CI: −0.95, −0.25). By contrast, the EAPC for mortality ASR was −0.80 (95% CI: −0.95, −0.64) in low-middle SDI regions and −0.37 (95% CI: −0.43, −0.31) in low-SDI regions. Similarly, the EAPC for DALYs ASR peaked in low-SDI regions at −0.32 (95% CI: −0.41, −0.22) and was lowest in high-middle SDI regions at −1.13 (95% CI: −1.31, −0.95). From 1990 to 2021, incidence and prevalence of ASRs increased in all SDI categories except the low-middle SDI group, where incidence of ASR declined from 0.38 (95% UI: 0.25, 0.44) to 0.37 (95% UI: 0.32, 0.46), with EAPCs as described above. Mortality and DALYs ASRs decreased steadily across all SDI areas. Regionally, East and South Asia and Central Europe saw sharp increases, while Southern Latin America, Western Europe, Australasia, and North Africa showed reductions. For mortality and DALYs, the greatest increases occurred in Oceania, Southern Sub-Saharan Africa, Southeast Asia, and Central Latin America, while North Africa and the Middle East, Southern Latin America, Western Europe, and Australasia experienced the most notable declines (Table 1, Table 2, Tables S1 and S2, Figure 1 and Figure S1).

**Figure 2:**
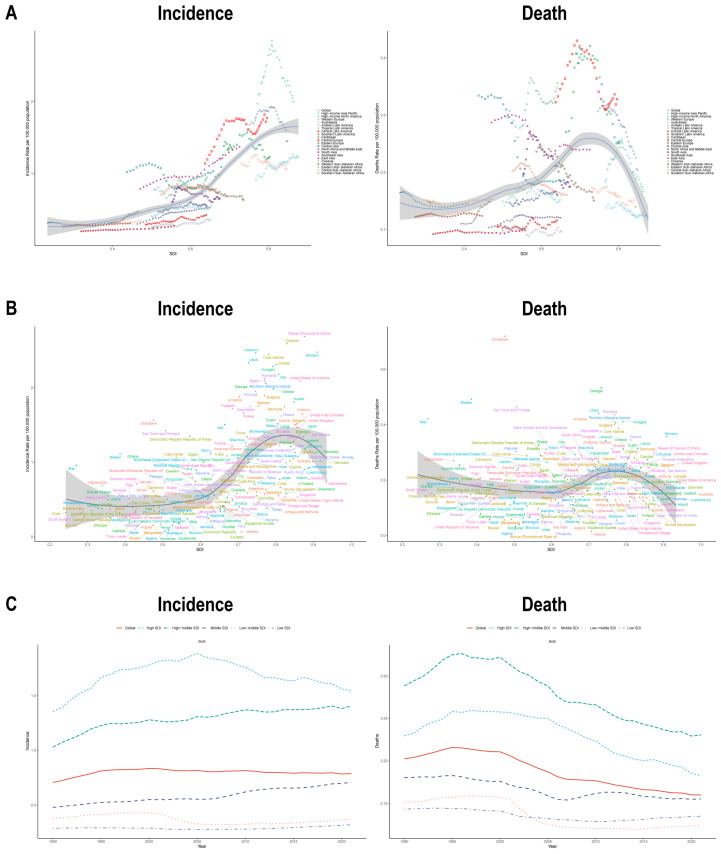
SDI-related global burden of disease: (**A**) Correlation between incidence (**left**) and mortality (**right**) with SDI across 27 GBD regions in 2021; (**B**) Relationship between incidence (**left**) and mortality (**right**) with SDI in 204 countries in 2021; (**C**) Temporal trends in incidence (**left**) and mortality (**right**) changes across SDI regions from 1990 to 2021.

To derive more precise insights, we systematically analyzed the relationship between SDI and EOBC burden across 21 regions and 204 countries. Among individuals aged 15–49 years, the correlation between SDI and incidence, mortality, prevalence, and DALYs followed an approximately S-shaped trajectory: as SDI increased, these indicators first declined slightly, then rose gradually to a peak, and finally declined rapidly. Regional analyses confirmed this nonlinear pattern, with middle-SDI regions bearing the heaviest burden (Figure 2A,B and Figure S2A,B). Temporal trend analysis revealed heterogeneous patterns: incidence increased slightly in high-middle and low-SDI groups, while the other three SDI groups exhibited an initial increase followed by a decline. In contrast, mortality trends showed consistent declines across all groups, albeit at varying rates (Figure 2C and Figure S2C).

**Figure 3:**
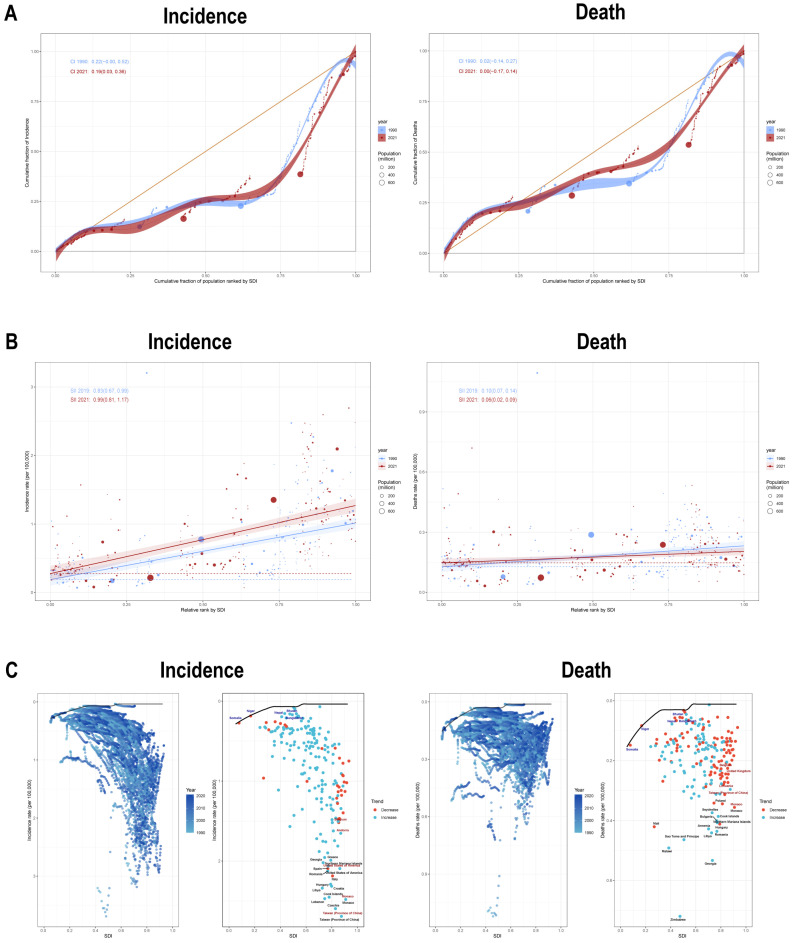
Global Inequalities in EOBC Burden (1990–2021). Health inequality metrics for incidence (**left**) and mortality (**right**) through regression curves (**A**) and concentration curves (**B**). (**C**) The efficiency frontiers for incidence and mortality are delineated by a solid black line, while individual countries/regions are represented by red data points. Temporal trends (1990–2021) are visualized through point coloring: red indicates growth rates during this period, while blue denotes reduction rates.

Our inequality analysis revealed pronounced socioeconomic disparities in the burden of EOBC. Between 1990 and 2021, the slope index of inequality (SII) changed markedly but remained positive. Specifically, the SII for incidence/prevalence increased from 0.83 (95% UI: 0.67, 0.99)/6.77 (95% UI: 5.57, 7.97) to 0.99 (95% UI: 0.81, 1.17)/8.50 (95% UI: 7.12, 9.88). In contrast, the SII for mortality/DALYs decreased from 0.10 (95% UI: 0.07, 0.14)/5.26 (95% UI: 3.37, 7.16) to 0.06 (95% UI: 0.02, 0.09)/2.95 (95% UI: 1.22, 4.69) (Figure 3A and Figure S3A). Concurrently, the concentration indices (CIs) for all indicators declined: the CI for incidence fell from 0.22 to 0.19, and for prevalence from 0.26 to 0.22, while both mortality and DALYs CIs decreased from 0.02 to 0.00. These findings indicate that from 1990 to 2021, the EOBC burden became more concentrated among populations with higher socioeconomic status, while overall health inequality has gradually decreased (Figure 3B and Figure S3B).

We constructed an SDI–ASR efficiency frontier (1990–2021) to quantify the achievable reduction in EOBC prevalence through optimal development. Based on 2021 data, we calculated the efficiency gaps of each country and territory relative to the theoretical frontier to estimate their untapped potential. Among 204 countries and territories, the 15 with the largest incidence efficiency gaps were Taiwan (Province of China), Czechia, Monaco, Lebanon, Cook Islands, Libya, Croatia, Hungary, Italy, Romania, the United States of America, Spain, Northern Mariana Islands, and Georgia; the prevalence distribution closely mirrored that of incidence (Figure 3C and Figure S3C).

The 15 regions with the largest mortality efficiency gaps were Zimbabwe, Georgia, Malawi, Sao Tome and Principe, Libya, Romania, Armenia, Hungary, Northern Mariana Islands, Bulgaria, Cook Islands, Mali, Seychelles, Monaco, and Poland. The DALYs distribution was also highly similar to that of mortality. These patterns suggest that the above regions may face specific public health challenges. Conversely, some countries—such as Niger, Nigeria, Somalia, Honduras, and Albania—were consistently close to the frontier across metrics. Frontier analysis showed that countries with the largest incidence and prevalence gaps were mainly in high- and high-middle SDI regions, while mortality and DALYs gaps were more geographically.

### 3.3. Demographic Characteristics of Early-Onset Bladder Cancer

**Figure 4:**
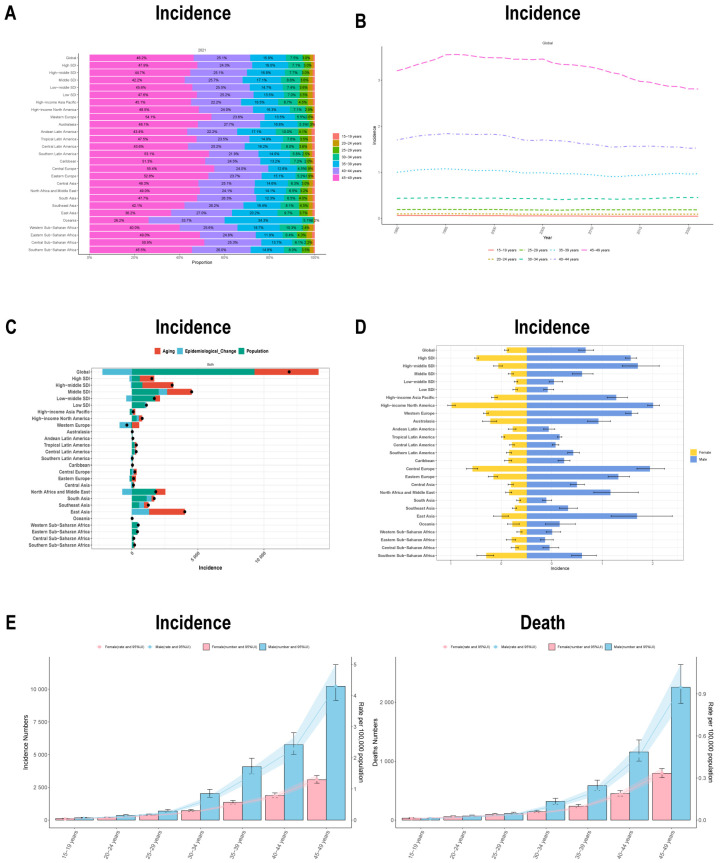
Age-specific Temporal, Spatial, and Gender Patterns. (**A**) Proportional age distribution of EOBC incidence; (**B**) Temporal trends in incidence stratified by age; (**C**) Decomposition analysis of contributions from aging, epidemiological changes, and population growth to global incidence trends; (**D**) Gender-stratified incidence across different regions; (**E**) Gender-stratified incidence (**left**) and mortality (**right**) across different age groups.

Age-stratified analysis revealed distinct spatiotemporal patterns in disease burden. Across countries and regions at all SDI classes, the 40–44 and 45–49 age groups were the most affected, indicating a modest shift in overall disease burden to middle-aged populations from the older one. The oldest subgroup (45–49 years) consistently possessed the highest share of incidence, mortality, prevalence, and DALYs across most geographic regions, followed by the 40–44 age group, while younger age groups maintained relatively low levels (Figure 4A and Figure S4A). Longitudinal assessment (1990–2021) showed that disease burden indicators in the older groups (40–44 and 45–49 years) generally declined, whereas those in the younger groups remained largely stable. This suggests the presence of age-specific differences in detection and management efficiency (Figure 4B and Figure S4B).

### 3.4. Decomposition of Global Burden

At the global and regional levels, changes in disease burden can be attributed to three factors: aging, epidemiological changes, and population growth. In most regions, population aging and growth have positively influenced incidence, mortality, prevalence, and DALYs. In high and high-middle SDI areas, aging is the dominant factor, whereas population growth has a greater impact in lower SDI regions. Epidemiological changes, however, have negatively affected disease burden metrics in Western Europe. In regions like the Caribbean and Oceania, the disease burden has remained largely unchanged (Figure 4C, Figures S4C, and S5C).

### 3.5. Age-Specific Spatiotemporal and Gender Patterns of Disease Burden

All disease burden metrics exhibited an age-dependent increase, peaking in the 45–49 age group for incidence, mortality, prevalence, and DALYs. Male predominance was consistent across all age groups, with the sex gap widening progressively with age. Among individuals >30 years, males demonstrated at least two-fold higher rates than females for all four metrics. These findings unequivocally demonstrate greater male susceptibility to EOBC (Figure 4E and Figure S5D,E).

### 3.6. Risk Analysis of Early-Onset Bladder Cancer Burden

**Figure 5:**
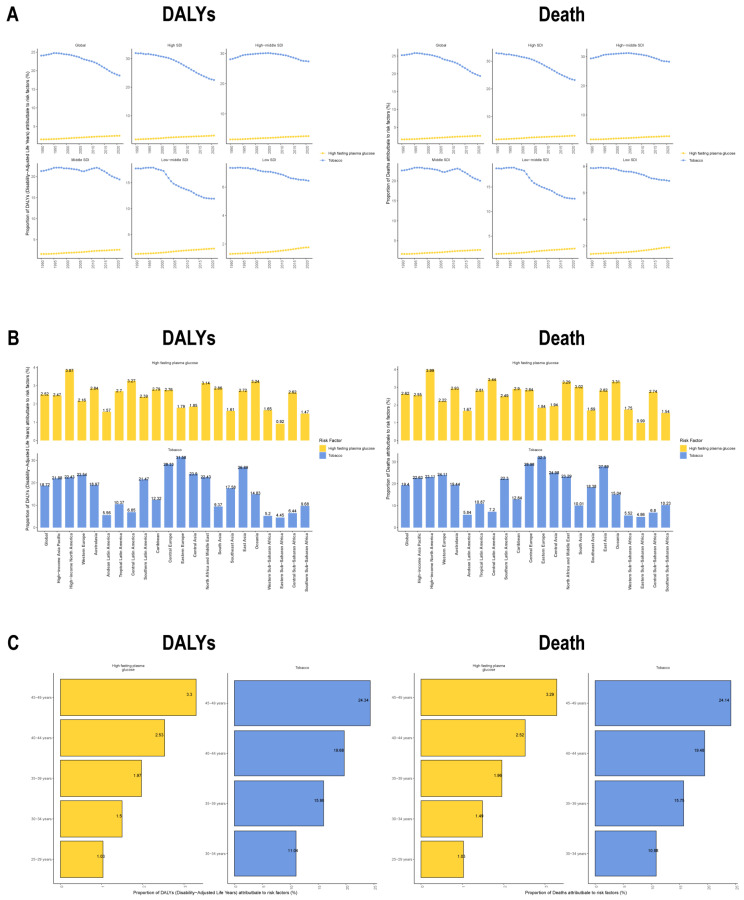
Risk factor analysis for early-onset bladder cancer. (**A**) Global and SDI-region-specific trends in risk factor contributions to DALYs (**left**) and mortality (**right**). (**B**) Risk factor contributions to DALYs (**left**) and mortality (**right**) across 21 regions. (**C**) Age-stratified trends in risk factor contributions to DALYs (**left**) and mortality (**right**). The two risk factors analyzed—high fasting plasma glucose (yellow) and smoking (blue)—are presented with distinct color coding.

Globally, among known risk factors, smoking accounted for the highest proportion of DALYs and mortality (18.55%), followed by high fasting plasma glucose (FPG) (2.44%). This ranking remained consistent across all SDI regions. Notably, from 1990 to 2021, the proportional contribution of smoking declined in all SDI regions, while that of FPG increased (Figure 5A). Among the 21 regions analyzed, smoking exhibited a similar distribution in its contributions to both DALYs and mortality, with the highest burdens observed in Eastern Europe and Central Europe and the lowest in Eastern Sub-Saharan Africa and Western Sub-Saharan Africa. Similarly, FPG showed parallel trends, with the greatest contributions in high-income North America and Central Latin America, and the smallest in Eastern Sub-Saharan Africa and Southern Sub-Saharan Africa (Figure 5B). Age-stratified analysis revealed that the proportional contributions of both smoking and FPG to DALYs and mortality increased with advancing age (Figure 5C).

These results demonstrate the dominant roles of smoking and FPG in EOBC DALYs and mortality burden, as well as their strong associations with socioeconomic development and aging. They further underscore the importance of targeted preventive measures.

### 3.7. AAPC and Age-Period-Cohort Analysis of Early-Onset Bladder Cancer

**Figure 6:**
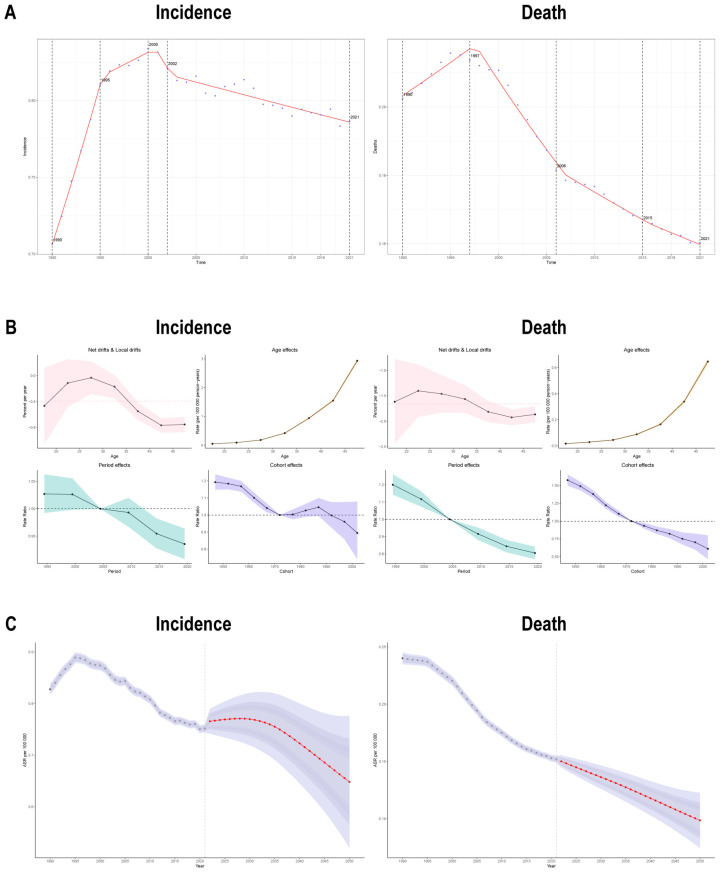
Joinpoint regression, Age-Period-Cohort modeling to estimate temporal patterns, and projected trends in early-onset bladder cancer burden. (**A**) Temporal trend plots showing AAPC trajectories for incidence (**left**) and mortality (**right**) between 1990 and 2021; (**B**) APC decomposition with: (i) Net/local drift greater than 0 indicating burden escalation (**upper left**), (ii) Age-specific effect curves (**upper right**), and (iii) Period/cohort rate ratios > 1 indicating elevated risk (**lower panel**); (**C**) Age-standardized rate (ASR) projections for incidence (**left**) and mortality (**right**).

Using joinpoint regression analysis, we identified distinct temporal patterns in early-onset bladder cancer burden. The incidence trajectory exhibited four phases: initial rapid growth (1990–1995; APC = 2.794), followed by decelerated growth to peak (1995–2000; APC = 0.390), a brief rapid decline (2000–2002; APC = −1.239), and finally a gradual decline (2002–2021; APC = −0.204). Mortality showed early growth to peak (1990–1997; APC = 0.937), followed by a rapid decline (1997–2006; APC = −2.015), and subsequently two phases of diminishing decline rates (2006–2015; APC = −0.936) and (2015–2021; APC = −0.712). Notably, DALYs demonstrated highly consistent trends with mortality. While prevalence mirrored incidence in the early period, it diverged later with a gradual decline (2000–2019) followed by an accelerated decline (2019–2021). The AAPC values were 0.353 for incidence, −0.768 for mortality, 0.556 for prevalence, and −0.760 for DALYs (Figure 6A and Figure S6A).

Age-period-cohort analysis revealed significant net drift trends, with incidence (−0.3935896), mortality (−1.657328), prevalence (−0.2139411), and DALYs (−1.59725) all showing significant downward trends (all *p* < 0.05). Age effects demonstrated consistently increasing impacts across all metrics. Period effects showed similar patterns—incidence; mortality; prevalence; and DALYs rate ratios (RRs) remained above 1.0 before 2005 but consistently below 1.0 thereafter, reflecting sustained improvements in disease burden. Cohort effects displayed slightly different patterns: incidence and prevalence RRs remained below 1.0 after 1992 and 1997, respectively, while mortality and DALYs RRs fell below 1.0 as early as 1972 (Figure 6B and Figure S6B).

### 3.8. Global Disease Burden Prediction for Early-Onset Bladder Cancer to 2050

We projected disease burden through 2050 using Bayesian age-period-cohort (BAPC) analysis. The BAPC model predicts that incidence and prevalence will continue to rise for approximately the next decade before stabilizing and declining, while mortality and DALYs will maintain their current downward trends. These projections reflect continuous progress in disease prevention, diagnosis, and treatment (Figure 6C and Figure S6C).

## 4. Discussion

This study provides a comprehensive global assessment of the burden of early-onset bladder cancer (EOBC). Its unique value lies not only in updating the descriptive epidemiology but also in applying structural decomposition and efficiency frontier analysis to explain the underlying drivers and evaluate the performance of health systems across development levels. Between 1990 and 2021, the number of EOBC cases increased significantly, with a rise in prevalence and deaths. Notably, the burden was heaviest in middle-SDI regions. Decomposition analysis revealed that in high-SDI countries, population aging was the main contributor to the increasing burden, while in low- and middle-SDI regions, population growth was the dominant factor [18]. This finding highlights the significant role of demographic transitions in shaping the trends of EOBC incidence. Efficiency frontier analysis identified significant gaps in incidence control in high-SDI countries (e.g., Italy and the United States). This is a key qualitative insight, revealing that many well-resourced systems are underperforming relative to their potential. Some low-SDI countries exhibited mortality rates close to the frontier, which may reflect delayed diagnosis and data underreporting rather than a genuinely lower burden. However, given the limitations of the GBD data, causal claims should be made cautiously, and further research is required to better understand these patterns. As the first study to apply structural decomposition and efficiency evaluation to EOBC, these findings not only quantify disparities in disease control performance across countries at different development levels but also provide empirical evidence to guide stratified prevention strategies and optimize global cancer control resource allocation.

Although EOBC accounts for a relatively small proportion of all early-onset cancers, its global incidence increased by over 60% from 1990 to 2021, with mortality and DALYs also showing upward trends, indicating a sustained and growing health threat among individuals aged 15–49 [2]. A pivotal new finding is the shift in the epicenter of the burden: Contrary to previous perceptions, we found that the EOBC burden is not predominantly concentrated in high-SDI countries but middle-SDI regions instead [6]. In contrast, low-SDI countries—characterized by younger population structures and limited diagnostic capacity—exhibit low incidence yet only modest declines in mortality. This pattern may reflect challenges in healthcare access and diagnostic limitations rather than a genuine lower burden [3]. This triad of patterns—rising burden in middle-SDI, persistent high mortality in low-SDI, and efficiency gaps in high-SDI—constitutes a novel characterization of global EOBC inequality. Further research is needed to explore these potential contributing factors. This dual structural inequality has rarely been quantitatively demonstrated in prior literature. Such cross-regional developmental disparities not only exacerbate global health inequities but also underscore the persistent gap in current cancer control strategies for the adolescent and young adult (AYA) population [1,8,12].

Through three-dimensional decomposition of EOBC burden changes, we quantified the contributions of population aging, epidemiologic changes, and population growth to the global and regional trends from 1990 to 2021. In high and high-middle regions, population aging contributed most substantially to the increases in EOBC incidence, mortality, prevalence, and DALYs; in contrast, population growth remained the dominant driver in other lower SDI regions, consistent with previous epidemiological research [19]. This manifests the fundamental role of demographic transitions in shaping EOBC epidemiological patterns. We also found that in certain high-income regions, such as Western Europe, epidemiologic changes exerted a negative contribution to disease burden, and the reductions are likely due to improvements in coding, changes in smoking prevalence, or a shift in age distribution, rather than being directly attributed to early screening. However, minimal indicator improvements were observed in the Caribbean and some Oceanian countries, reflecting delays in public health responsiveness. Taken together, the decomposition analysis provides the mechanistic explanation for the observed geographic shift in burden, which is a core qualitative contribution of this study [20].

Additionally, the EOBC burden is progressively shifting toward individuals aged 40–49, who bear the highest incidence rates and DALYs in most countries [3]. Moreover, this study reveals notable sex disparities, with male incidence and mortality rates generally exceeding those of females by more than twofold, and the gap widening with age. Potential explanations include differences in smoking prevalence, occupational exposure patterns, sex hormone metabolism, urinary tract anatomy, and genetic susceptibility [21,22,23]. Notably, in Eastern and Southern Sub-Saharan Africa, this sex gap narrows, suggesting the influence of region-specific risk factors such as schistosomiasis [24,25]. Recent research also indicates that certain molecular subtypes and immune microenvironment characteristics may differ by sex, thereby influencing both disease risk and prognosis [22]. Targeted early screening and risk-reduction strategies for high-risk male populations should be prioritized in public health agendas.

Given the concentration of burden in the 40–49 age band, we considered whether redefining EOBC (e.g., as 20–49 or 25–49 years) would alter our core conclusions. While absolute estimates would vary, the key trends and inequalities we identify are likely robust. The dominant demographic drivers (aging vs. population growth), the geographic shift to middle-SDI regions, and the identified efficiency gaps are all structural phenomena tied to broader development levels and health system factors, not sensitive to the precise upper age limit within the young adult spectrum. Thus, the imperative for stratified, demographically informed prevention remains unchanged.

Globally, smoking remains the leading attributable risk factor for EOBC mortality and DALYs, accounting for over 25% of the burden in Central and Eastern Europe [26]. Although smoking-related attribution has declined in most countries, a sustained increase was observed in the proportion of burden attributable to hyperglycemia, particularly in areas with high obesity prevalence like North America and Latin America [27]. This trend is highly consistent with recent studies linking metabolic syndrome to urological cancers [28,29,30]. This signals an important and emerging shift in the risk factor landscape for EOBC, necessitating an expanded prevention paradigm. Establishing early cancer warning systems for individuals with diabetes could substantially reduce the disease burden in the medium to long term [19,31]. It is important to note, however, that hyperglycemia (high FPG) is an indirect proxy for metabolic syndrome. This metric does not fully capture other critical components of metabolic syndrome, such as abdominal obesity or insulin resistance, which may also contribute to EOBC. Moreover, many relevant EOBC risk factors, particularly those in low-income, schistosomiasis-endemic regions, are not captured by the available data, which could lead to an underestimation of the actual disease burden in these areas.

The BAPC model projections show that EOBC incidence and prevalence will continue to rise over the next decade, followed by a gradual stabilization and decline after 2030, whereas mortality and DALYs are expected to maintain a downward trajectory but with only modest reductions overall. This pattern is closely associated with recent advances in high-income countries in surgical techniques, immunotherapy, and comprehensive management strategies [31,32,33]. The increasing burden of EOBC has significant economic implications. High treatment costs and long-term care for young cancer patients, particularly in high-income countries, add substantial financial pressure on healthcare systems. Similarly, low-SDI countries with limited healthcare access may face exacerbated economic challenges due to late-stage diagnosis and higher treatment costs [34,35]. While these findings suggest that current control measures have yielded tangible benefits, they also underscore that the progress achieved remains insufficient to fully reverse the trend. The model further reminds us to take intervention measures to prevent the overall rate of global burden reduction from being limited [1,36].

This study methodologically innovates by incorporating structural decomposition modeling and efficiency frontier analysis into EOBC research, surpassing the descriptive trend statistics prevalent in existing literature to uncover the underlying mechanisms of disease burden [5,19,37]. By integrating SDI, gender, and age heterogeneity dimensions, we propose feasible intervention strategies tailored to specific countries and populations. Nevertheless, several limitations should be acknowledged. First, GBD estimates depend heavily on modeling, and data underreporting remains possible in low-SDI countries. Second, the risk factor analysis was constrained by measurable variables, excluding region-specific exposures such as occupational hazards and parasitic infections [24]. Thirdly, the frontier model is based on a static SDI assumption, and future socioeconomic shifts that may reshape disease burden require further simulation-based exploration. Finally, the age threshold used to define EOBC may not be universally applicable across all countries, suggesting that policy design should be adapted to local epidemiological contexts to ensure optimal intervention outcomes.

Using GBD 1990–2021 data, this study comprehensively delineates the long-term trends, structural inequalities, and driving mechanisms of EOBC among individuals aged 15–49 years worldwide. For the first time in this field, decomposition analysis and SDI-efficiency frontier evaluation were applied, revealing substantial disparities in disease control performance and resource utilization across countries at different development levels. The results indicate that the EOBC burden has continued to rise globally, with a particularly marked increase in middle-SDI countries. Regional variations in burden change were jointly driven by demographic aging, epidemiological changes, and population growth. Smoking remains the predominant modifiable risk factor, while the contribution of metabolic risk factors is increasing. Frontier analysis identified notable efficiency gaps in high-SDI countries, underscoring the urgent need to optimize public health initiatives and resource allocation. This study not only provides panoramic evidence of EOBCs global epidemiological patterns but also offers methodological and policy-relevant insights to support tiered prevention, targeted interventions, and the development of international collaborative frameworks.

### 4.1. Highlights

The unique contribution of this work is threefold: (1) it explains global burden trends through the prism of differential demographic drivers, (2) it introduces a performance efficiency metric to evaluate health system response, and (3) it integrates these analyses to reveal a “dual inequality” in EOBC burden and control. These findings provide not just updated statistics but also a refined conceptual and strategic framework for tiered prevention, arguing for interventions that are demographically informed, performance-targeted, and risk-adapted. The study highlights the growing burden of EOBC, particularly in middle-SDI countries, emphasizing the need for early detection and risk factor modification. Public health strategies should prioritize screening, especially in low- and middle-income regions, and focus on modifiable risk factors such as smoking and metabolic disorders. Improving healthcare systems and diagnostic capabilities in low-SDI countries is essential to reduce missed diagnosis opportunities.

### 4.2. Limitations

This study relies on GBD data, which may be limited by underreporting in low-SDI countries. Future research should incorporate more localized data and dynamic models to account for changing socioeconomic factors and their impact on the EOBC burden. Further studies are needed to refine the understanding of these factors and explore ways to mitigate the growing global burden of EOBC.

## 5. Conclusions

This study relies on GBD data, which may be limited by underreporting in low-SDI countries. Future research should incorporate more localized data and dynamic models to account for changing socioeconomic factors and their impact on the EOBC burden. Further studies are needed to refine the understanding of these factors and explore ways to mitigate the growing global burden of EOBC. This study provides the most comprehensive evaluation to date of the global burden, temporal trends, and structural drivers of early-onset bladder cancer (EOBC). By integrating three-dimensional decomposition analysis with SDI-ASR efficiency frontier modeling, we identified marked disparities in disease dynamics and control performance across development levels. From 1990 to 2021, EOBC incidence and prevalence rose substantially worldwide, with the heaviest burden concentrated in middle-SDI regions, while mortality and DALYs declined only modestly. Demographic transitions—particularly population aging in high-SDI and high-middle-SDI settings and population growth in lower-SDI regions—emerged as dominant drivers, alongside persistent sex differences and shifting age patterns toward individuals aged 40–49 years. Smoking remains the leading modifiable risk factor, but the rising contribution of metabolic disorders underscores the need for integrated prevention strategies. While the increasing role of metabolic risk factors, particularly high fasting plasma glucose (FPG), in the burden of early-onset bladder cancer (EOBC) is evident, it is important to emphasize that FPG is an indirect proxy for metabolic syndrome. This metric does not fully capture other critical components, such as abdominal obesity and insulin resistance, which may also contribute to EOBC. Additionally, many relevant EOBC risk factors, especially those specific to low-income, schistosomiasis-endemic regions, are not included in the Global Burden of Disease (GBD) data, which could lead to an underestimation of the actual disease burden in these regions. Our findings highlight substantial efficiency gaps even in resource-rich settings and reveal missed opportunities for timely diagnosis in low-SDI countries. These results provide robust, evidence-based guidance for prioritizing targeted screening, risk-factor modification, and resource allocation, and offer a methodological framework applicable to other early-onset cancers in the era of evolving global demographics.

## Figures and Tables

**Table 1:** Numbers of Incidence, Incidence ASR, and EAPC Incidence ASR in 1990 and 2021 from Global Disease Burden 2021.

Location	Number of Incidence		Incidence of ASR			EAPC Incidence ASR
	1990	2021	1990	2021	PC	EAPCs
Global	19,146.94 (16,119.05, 20,578.77)	31,053.76 (28,341.92, 34,322.02)	0.71 (0.59, 0.76)	0.79 (0.72, 0.87)	11.33 (−1.22, 35.86)	0.09 (−0.06, 0.23)
SDI Regions						
High SDI	6237.46 (6041.29, 6401.42)	7749.97 (7474.13, 8089.56)	1.35 (1.31, 1.39)	1.54 (1.49, 1.61)	14.01 (9.13, 20.25)	0.15 (−0.17, 0.48)
High-middle SDI	5801.57 (4751.31, 6423.84)	8855.59 (7836.71, 10,203.65)	1.03 (0.84, 1.14)	1.41 (1.24, 1.62)	36.84 (14.64, 68.75)	0.77 (0.64, 0.90)
Middle SDI	4361.02 (3364.87, 4917.80)	8886.95 (7730.48, 10,264.97)	0.48 (0.37, 0.54)	0.71 (0.62, 0.82)	47.85 (21.67, 95.67)	1.22 (1.13, 1.30)
Low-middle SDI	2089.67 (1354.66, 2429.03)	3792.58 (3274.51, 4724.48)	0.38 (0.25, 0.44)	0.37 (0.32, 0.46)	−1.58 (−22.47, 64.58)	−0.71 (−1.03, −0.39)
Low SDI	637.56 (546.33, 741.69)	1740.07 (1439.00, 2103.83)	0.29 (0.25, 0.34)	0.32 (0.27, 0.39)	11.23 (−8.04, 34.12)	0.17 (0.04, 0.30)
GBD Geographic Regions						
East Asia	5439.65 (3588.00, 6462.72)	9447.24 (7566.05, 11,984.10)	0.79 (0.52, 0.94)	1.37 (1.10, 1.74)	73.76 (27.38, 168.47)	1.64 (1.49, 1.79)
Southeast Asia	714.92 (598.01, 815.54)	1965.28 (1632.10, 2326.94)	0.30 (0.25, 0.34)	0.53 (0.44, 0.63)	75.40 (43.82, 114.42)	1.60 (1.37, 1.82)
Oceania	9.92 (6.10, 13.52)	33.40 (19.34, 46.85)	0.31 (0.19, 0.42)	0.47 (0.27, 0.66)	52.15 (15.79, 108.00)	1.48 (1.40, 1.55)
Central Asia	200.90 (177.68, 225.35)	319.90 (277.18, 367.60)	0.60 (0.53, 0.68)	0.66 (0.57, 0.75)	8.90 (−8.72, 30.88)	−0.39 (−0.72, −0.07)
Central Europe	708.46 (671.70, 746.42)	934.20 (840.73, 1029.72)	1.14 (1.08, 1.20)	1.77 (1.60, 1.95)	55.40 (39.45, 72.33)	0.83 (0.46, 1.20)
Eastern Europe	1054.56 (984.21, 1171.06)	1187.93 (1078.02, 1304.56)	0.96 (0.89, 1.06)	1.23 (1.12, 1.36)	29.11 (13.04, 45.93)	0.26 (−0.19, 0.71)
High-income Asia Pacific	854.88 (801.88, 911.67)	956.45 (878.18, 1047.16)	0.92 (0.86, 0.98)	1.22 (1.12, 1.34)	32.77 (19.32, 46.02)	0.82 (0.69, 0.96)
Australasia	122.99 (111.72, 134.17)	154.61 (136.22, 174.78)	1.14 (1.04, 1.24)	1.07 (0.94, 1.21)	−6.06 (−18.73, 10.16)	−0.24 (−0.40, −0.08)
Western Europe	3112.01 (3000.98, 3219.99)	2735.39 (2609.80, 2868.26)	1.61 (1.55, 1.66)	1.45 (1.38, 1.52)	−9.81 (−14.83, −4.34)	−0.30 (−0.59, −0.01)
Southern Latin America	187.33 (173.51, 204.88)	219.96 (198.40, 244.39)	0.76 (0.71, 0.84)	0.63 (0.57, 0.70)	−17.10 (−26.84, −6.00)	−0.77 (−0.90, −0.64)
High-income North America	2585.58 (2521.67, 2655.40)	3369.89 (3253.27, 3505.01)	1.73 (1.69, 1.78)	2.00 (1.93, 2.08)	15.16 (10.87, 19.31)	0.18 (−0.33, 0.69)
Caribbean	86.98 (80.71, 93.97)	131.83 (112.58, 152.95)	0.48 (0.44, 0.51)	0.55 (0.47, 0.64)	15.62 (−0.87, 35.61)	0.63 (0.48, 0.79)
Andean Latin America	45.76 (40.27, 52.31)	124.67 (99.71, 154.25)	0.25 (0.22, 0.28)	0.36 (0.29, 0.44)	45.16 (12.23, 85.75)	1.01 (0.82, 1.20)
Central Latin America	239.40 (231.24, 247.99)	570.03 (510.55, 633.88)	0.29 (0.28, 0.30)	0.43 (0.38, 0.48)	46.00 (29.29, 63.71)	1.08 (0.92, 1.24)
Tropical Latin America	322.72 (307.05, 338.43)	666.72 (636.25, 699.77)	0.41 (0.39, 0.43)	0.56 (0.53, 0.58)	35.37 (27.32, 44.13)	0.75 (0.54, 0.97)
North Africa and Middle East	1643.99 (986.45, 1975.41)	3456.77 (2827.74, 4400.30)	1.03 (0.62, 1.23)	1.03 (0.85, 1.32)	0.80 (−26.01, 87.95)	−0.55 (−0.96, −0.13)
South Asia	1054.05 (875.06, 1218.17)	2747.01 (2327.97, 3318.50)	0.20 (0.17, 0.23)	0.27 (0.23, 0.33)	36.95 (11.61, 75.42)	0.82 (0.73, 0.92)
Central Sub-Saharan Africa	71.97 (54.99, 90.42)	222.59 (166.84, 288.64)	0.29 (0.23, 0.37)	0.34 (0.26, 0.44)	15.82 (−17.11, 59.09)	0.44 (0.26, 0.62)
Eastern Sub-Saharan Africa	264.21 (227.15, 309.60)	690.67 (533.36, 908.85)	0.32 (0.27, 0.37)	0.33 (0.25, 0.43)	4.13 (−21.53, 38.85)	−0.14 (−0.30, 0.03)
Southern Sub-Saharan Africa	200.79 (171.74, 224.14)	408.94 (345.65, 480.59)	0.78 (0.67, 0.87)	0.95 (0.80, 1.11)	21.52 (0.04, 46.27)	0.55 (0.27, 0.84)
Western Sub-Saharan Africa	225.87 (180.49, 277.82)	710.28 (558.34, 917.70)	0.26 (0.21, 0.32)	0.31 (0.24, 0.40)	17.40 (−8.04, 49.80)	0.48 (0.41, 0.54)

**Table 2:** Numbers of Deaths, Death ASR, and EAPC Death ASR in 1990 and 2021 from Global Disease Burden 2021.

Location	Number of Deaths		Death ASR			EAPC Death ASR
	1990	2021	1990	2021	PC	EAPCs
Global	5486.52 (4460.69,5976.30)	6327.78 (5732.75,6995.74)	0.20 (0.16,0.22)	0.16 (0.15,0.18)	−20.83 (−30.79,0.31)	−0.77 (−0.89, −0.65)
SDI Regions						
High SDI	1057.44 (1010.44, 1088.20)	917.76 (875.23, 961.49)	0.23 (0.22, 0.24)	0.18 (0.17, 0.19)	−20.36 (−24.15, −14.94)	−0.48 (−0.69, −0.27)
High-middle SDI	1627.90 (1284.87, 1829.24)	1450.37 (1283.46, 1669.82)	0.29 (0.23, 0.32)	0.23 (0.20, 0.27)	−20.13 (−33.78, 1.85)	−0.68 (−0.87, −0.50)
Middle SDI	1642.49 (1268.47, 1852.70)	1952.80 (1721.06, 2255.05)	0.18 (0.14, 0.20)	0.16 (0.14, 0.18)	−13.74 (−28.79, 14.19)	−0.60 (−0.68, −0.53)
Low-middle SDI	835.94 (580.74, 959.86)	1266.92 (1093.42, 1543.63)	0.15 (0.11, 0.17)	0.12 (0.11, 0.15)	−17.81 (−34.10, 25.09)	−0.80 (−0.95, −0.64)
Low SDI	317.17 (275.85, 370.58)	733.84 (609.11, 890.46)	0.14 (0.12, 0.17)	0.14 (0.11, 0.16)	−5.71 (−22.58, 14.61)	−0.37 (−0.43, −0.31)
GBD Geographic Regions						
East Asia	1976.61 (1300.19, 2354.64)	1648.81 (1311.75, 2102.16)	0.29 (0.19, 0.34)	0.24 (0.19, 0.31)	−16.54 (−39.51, 29.80)	−0.78 (−0.92, −0.65)
Southeast Asia	244.86 (208.81, 278.16)	447.42 (376.01, 539.97)	0.10 (0.09, 0.12)	0.12 (0.10, 0.15)	16.59 (−5.02, 42.82)	0.53 (0.42, 0.64)
Oceania	3.50 (2.04, 4.75)	10.45 (5.97, 14.94)	0.11 (0.06, 0.15)	0.15 (0.08, 0.21)	34.72 (2.54, 84.54)	0.88 (0.78, 0.98)
Central Asia	65.61 (58.06, 74.11)	85.46 (73.75, 98.82)	0.20 (0.17, 0.22)	0.18 (0.15, 0.20)	−10.92 (−25.62, 8.01)	−0.63 (−0.93, −0.34)
Central Europe	200.25 (191.20, 208.97)	172.72 (158.00, 189.41)	0.32 (0.31, 0.34)	0.33 (0.30, 0.36)	1.65 (−7.17, 10.92)	0.07 (−0.23, 0.36)
Eastern Europe	298.15 (277.47, 330.21)	244.78 (219.93, 271.48)	0.27 (0.25, 0.30)	0.25 (0.23, 0.28)	−5.90 (−18.73, 7.30)	0.11 (−0.38, 0.59)
High-income Asia Pacific	128.51 (119.27, 136.78)	96.03 (89.99, 103.39)	0.14 (0.13, 0.15)	0.12 (0.12, 0.13)	−11.32 (−18.81, −3.33)	−0.67 (−0.77, −0.57)
Australasia	18.74 (17.14, 20.41)	20.16 (17.98, 22.60)	0.17 (0.16, 0.19)	0.14 (0.12, 0.16)	−19.63 (−30.92, −5.93)	−0.58 (−0.66, −0.51)
Western Europe	593.20 (575.38, 610.99)	384.44 (368.06, 403.36)	0.31 (0.30, 0.32)	0.20 (0.20, 0.21)	−33.51 (−36.84, −29.92)	−0.86 (−1.02, −0.70)
Southern Latin America	57.82 (53.46, 62.64)	52.26 (47.13, 57.79)	0.24 (0.22, 0.26)	0.15 (0.14, 0.17)	−36.19 (−43.51, −28.06)	−1.50 (−1.60, −1.40)
High-income North America	273.33 (266.42, 281.01)	280.95 (271.49, 292.16)	0.18 (0.18, 0.19)	0.17 (0.16, 0.17)	−9.18 (−12.60, −5.86)	0.28 (−0.08, 0.65)
Caribbean	26.84 (24.33, 29.66)	38.20 (31.72, 45.95)	0.15 (0.13, 0.16)	0.16 (0.13, 0.19)	8.56 (−8.04, 28.20)	0.45 (0.39, 0.52)
Andean Latin America	18.61 (16.36, 21.36)	33.87 (27.03, 42.40)	0.10 (0.09, 0.11)	0.10 (0.08, 0.12)	−3.03 (−25.73, 21.32)	−0.27 (−0.38, −0.17)
Central Latin America	85.66 (83.12, 88.67)	154.69 (137.34, 171.93)	0.10 (0.10, 0.11)	0.12 (0.10, 0.13)	10.72 (−2.52, 23.48)	0.04 (−0.10, 0.17)
Tropical Latin America	117.28 (111.79, 122.66)	190.61 (182.07, 200.13)	0.15 (0.14, 0.16)	0.16 (0.15, 0.17)	6.50 (0.18, 12.87)	0.09 (−0.05, 0.22)
North Africa and Middle East	535.78 (320.25, 639.52)	623.42 (516.97, 786.17)	0.33 (0.20, 0.40)	0.19 (0.15, 0.24)	−44.22 (−58.28, 3.20)	−2.27 (−2.55, −1.99)
South Asia	486.58 (406.57, 560.64)	1001.08 (848.07, 1215.74)	0.09 (0.08, 0.11)	0.10 (0.08, 0.12)	8.12 (−11.68, 36.13)	0.22 (0.16, 0.28)
Central Sub-Saharan Africa	36.83 (28.32, 46.17)	99.80 (74.47, 130.91)	0.15 (0.12, 0.19)	0.15 (0.11, 0.20)	1.46 (−27.73, 42.57)	−0.21 (−0.32, −0.11)
Eastern Sub-Saharan Africa	136.96 (117.61, 161.44)	305.01 (235.65, 400.92)	0.16 (0.14, 0.19)	0.15 (0.11, 0.19)	−11.29 (−33.53, 19.35)	−0.58 (−0.65, −0.50)
Southern Sub-Saharan Africa	76.80 (66.51, 85.55)	153.61 (127.44, 184.25)	0.30 (0.26, 0.33)	0.36 (0.30, 0.43)	19.33 (−3.60, 46.54)	0.19 (−0.01, 0.40)
Western Sub-Saharan Africa	104.60 (84.29, 126.22)	284.02 (224.80, 364.64)	0.12 (0.10, 0.15)	0.12 (0.10, 0.16)	1.36 (−21.50, 28.68)	0.06 (0.02, 0.10)

## Data Availability

All data were accessed and downloaded via the Global Health Data Exchange (GHDx) platform: http://ghdx.healthdata.org/gbd-results-tool (accessed on 27 October 2025).

## Supplementary Materials

The following supporting information can be downloaded at https://www.mdpi.com/article/10.3390/healthcare14020193/s1, Table S1: Numbers of Prevalence, Prevalence ASR, EAPC Prevalence ASR in 1990 and 2021 from Global Disease Burden 2021. Table S2: Numbers of DALYs, DALYs ASR, EAPC DALYs ASR in 1990 and 2021 from Global Disease Burden 2021. Figure S1: Global distribution of early-onset bladder cancer burden in 2021. (A) Global maps showing EAPCs for prevalence (left) and DALYs (right). (B) Regional maps illustrating the same EAPC data. (C) Comparison of ASRs for prevalence (left) and DALYs (right) across GBD regions in 1990 versus 2021. Figure S2: Association between socio-demographic index (SDI) and early-onset bladder cancer burden. (A) Relationship between SDI and prevalence (left) and DALYs (right) across GBD regions. (B) Country-level association between SDI and prevalence (left) and DALYs (right). (C) Temporal trends of prevalence (left) and DALYs (right) stratified by SDI level from 1990 to 2021. Figure S3: Health inequality metrics for prevalence (left) and DALYs (right) through regression curves (A) and concentration curves (B). (C) The efficiency frontiers for prevalence and DALYs. Figure S4: Age-group composition and temporal trends of early-onset bladder cancer burden. (A) Proportional distribution of prevalence (left) and DALYs (right) by age group in 2021. (B) Temporal trends of prevalence (left) and DALYs (right) by age group from 1990 to 2021. (C) Decomposition of changes in prevalence (left) and DALYs (right) into aging, epidemiological change, and population growth. Figure S5: Mortality patterns, decomposition analysis, and age- and sex-specific burden of early-onset bladder cancer. (A) Proportional distribution of deaths by age group in 2021. (B) Temporal trends of mortality by age group from 1990 to 2021. (C) Decomposition analysis of changes in deaths by contributing factors. (D) Age-specific effects of mortality rates derived from the age–period–cohort model. (E) Sex-specific prevalence (left) and DALYs (right) by age group. Figure S6: Temporal trends and projections of early-onset bladder cancer burden. (A) Temporal trend plots analysis showing AAPC trajectories for prevalence (left) and DALYs (right). (B) APC analysis of prevalence (left) and DALYs (right). (C) Projected ASRs of prevalence (left) and DALYs (right) to 2050.

## Abbreviations

The following abbreviations are used in this manuscript:

AAPC	average annual percentage change
APC	annual percentage change
ASR	age-standardized rate
DALYs	disability-adjusted life years
EAPC	estimated annual percentage change
EOCs	early-onset cancers
EOBC	early-onset bladder cancer
GBD	Global Burden of Disease
SII	Slope Index of Inequality
CI	Concentration Index
SDI	socio-demographic index
UI	uncertainty interval
