# Supplementary material for: Unequal Progress in Early-Onset Bladder Cancer Control: Global Trends, Socioeconomic Disparities, and Policy Efficiency from 1990 to 2021
Source: Healthcare (Basel). 2026 Jan 12;14(2):193. doi: 10.3390/healthcare14020193 (PMC12840968; doi:10.3390/healthcare14020193)
Supplement: Supplementary file 1 [file healthcare-14-00193-s001.zip › Table S1.pdf]

Location	Number of Prevalence		Prevalence ASR			EAPC prevalence ASR
	1990	2021	1990	2021	PC	EAPCs
Global	138140.03(118326.12,147695.35)	239183.73(218878.34,264625.28)	5.10(4.37,5.45)	6.06(5.54,6.70)	18.85(6.10,43.78)	0.31(0.16,0.47)
**SDI Regions**						
High SDI	49306.48(47780.56,50656.21)	63458.12(61189.74,66191.68)	10.70(10.37,10.99)	12.64(12.18,13.18)	18.10(13.28,24.25)	0.28(-0.05,0.61)
High-middle SDI	42190.37(35041.88,46467.91)	70598.83(62580.19,81428.23)	7.48(6.21,8.23)	11.21(9.94,12.93)	50.01(26.90,82.94)	1.11(0.99,1.24)
Middle SDI	29201.16(22796.91,32905.65)	67820.30(58855.93,78724.27)	3.21(2.50,3.61)	5.40(4.69,6.27)	68.51(39.40,121.01)	1.65(1.58,1.72)
Low-middle SDI	13595.93(8714.78,15932.25)	26028.68(22457.18,32379.11)	2.47(1.58,2.89)	2.56(2.21,3.19)	3.82(-18.56,79.63)	-0.60(-0.95,-0.25)
Low SDI	3704.66(3171.42,4307.94)	11058.98(9172.90,13328.58)	1.68(1.43,1.95)	2.04(1.69,2.46)	21.66(1.45,45.79)	0.50(0.35,0.65)
**GBD Geographic Regions**						
East Asia	36886.83(24575.20,43754.14)	74812.96(60457.32,94226.10)	5.35(3.57,6.35)	10.87(8.78,13.69)	102.92(49.72,207.31)	0.78(0.68,0.88)
Southeast Asia	4956.73(4160.39,5642.25)	14926.97(12432.18,17713.29)	2.10(1.76,2.38)	4.03(3.35,4.78)	92.15(58.02,132.67)	0.05(0.02,0.08)
Oceania	67.21(43.54,92.03)	235.39(140.13,326.92)	2.10(1.36,2.88)	3.33(1.98,4.62)	58.17(21.11,112.56)	-0.48(-0.54,-0.42)
Central Asia	1425.85(1269.94,1602.09)	2386.52(2069.75,2734.85)	4.28(3.81,4.80)	4.89(4.24,5.61)	14.47(-3.33,36.56)	-0.85(-0.98,-0.72)
Central Europe	5073.01(4826.91,5354.41)	7265.19(6570.30,7984.74)	8.17(7.77,8.62)	13.79(12.47,15.15)	68.78(51.91,87.54)	-1.54(-1.66,-1.43)
Eastern Europe	7567.99(7075.96,8374.72)	9022.18(8235.92,9841.35)	6.86(6.42,7.59)	9.38(8.56,10.23)	36.64(20.55,53.34)	-0.61(-0.72,-0.51)
High-income Asia Pacific	6984.35(6555.17,7424.66)	8046.09(7408.81,8807.27)	7.52(7.06,8.00)	10.29(9.47,11.26)	36.71(23.43,49.85)	-1.24(-1.45,-1.04)
Australasia	985.07(896.30,1074.41)	1259.43(1115.16,1423.49)	9.13(8.31,9.96)	8.72(7.72,9.86)	-4.46(-17.74,12.07)	-0.66(-0.78,-0.53)
Western Europe	24257.58(23429.82,25130.48)	22237.53(21199.71,23313.62)	12.54(12.11,12.99)	11.80(11.25,12.37)	-5.94(-10.90,-0.18)	-1.33(-1.41,-1.25)
Southern Latin America	1322.99(1225.22,1437.17)	1656.90(1499.52,1837.16)	5.40(5.00,5.87)	4.78(4.32,5.30)	-11.58(-21.86,0.02)	-1.46(-1.63,-1.29)
High-income North America	21204.11(20651.03,21758.85)	27900.33(26916.15,29014.51)	14.23(13.86,14.60)	16.54(15.96,17.20)	16.26(11.92,20.36)	-0.47(-0.60,-0.34)
Caribbean	616.61(573.70,662.59)	947.51(820.41,1089.90)	3.38(3.14,3.63)	3.96(3.43,4.55)	17.22(0.66,36.00)	-0.20(-0.27,-0.12)
Andean Latin America	297.46(262.71,339.90)	914.99(733.65,1129.38)	1.60(1.41,1.82)	2.62(2.10,3.23)	63.88(27.92,107.77)	-0.88(-0.97,-0.79)
Central Latin America	1639.23(1581.33,1707.09)	4166.61(3739.61,4636.69)	2.01(1.94,2.09)	3.13(2.81,3.48)	55.85(38.05,74.52)	-1.66(-1.88,-1.43)
Tropical Latin America	2195.59(2089.92,2310.58)	4835.49(4613.70,5081.97)	2.80(2.66,2.94)	4.04(3.85,4.24)	44.31(35.85,53.14)	-2.54(-2.82,-2.26)
North Africa and Middle East	11646.67(7068.46,13994.71)	27550.73(22733.76,34947.70)	7.27(4.41,8.73)	8.24(6.80,10.45)	13.40(-16.07,108.14)	-0.34(-0.41,-0.28)
South Asia	6402.51(5335.13,7360.82)	18015.72(15316.20,21621.72)	1.21(1.01,1.39)	1.79(1.52,2.15)	47.87(21.27,86.94)	-0.13(-0.22,-0.03)
Central Sub-Saharan Africa	412.10(316.38,511.60)	1390.12(1046.92,1796.30)	1.69(1.30,2.10)	2.13(1.61,2.76)	26.32(-8.42,73.27)	-0.50(-0.53,-0.46)
Eastern Sub-Saharan Africa	1499.04(1301.48,1748.11)	4336.71(3370.98,5703.06)	1.80(1.56,2.10)	2.07(1.61,2.72)	15.24(-11.93,51.86)	-0.56(-0.62,-0.51)
Southern Sub-Saharan Africa	1318.12(1133.21,1466.10)	2647.56(2258.16,3110.49)	5.12(4.40,5.69)	6.13(5.23,7.21)	19.84(-0.12,43.35)	-1.18(-1.38,-0.98)
Western Sub-Saharan Africa	1381.00(1108.72,1696.56)	4628.80(3650.81,5939.85)	1.61(1.30,1.98)	2.02(1.59,2.59)	25.13(-1.02,58.79)	-0.31(-0.36,-0.26)

Table S1. Numbers of Prevalence , Prevalence ASR, EAPC Prevalence ASR in 1990 and 2021 from Global Disease Burden 2021
