# Supplementary material for: Unequal Progress in Early-Onset Bladder Cancer Control: Global Trends, Socioeconomic Disparities, and Policy Efficiency from 1990 to 2021
Source: Healthcare (Basel). 2026 Jan 12;14(2):193. doi: 10.3390/healthcare14020193 (PMC12840968; doi:10.3390/healthcare14020193)
Supplement: Supplementary file 1 [file healthcare-14-00193-s001.zip › Table S2.pdf]

Location	Number of DALYs		DALYs ASR			EAPC DALYs ASR
	1990	2021	1990	2021	PC	EAPCs
Global	279788.72(227184.21,305124.17)	324037.67(292792.64,359679.82)	10.32(8.38,11.26)	8.21(7.42,9.11)	-20.50(-30.49,0.48)	-1.05(-1.18,-0.93)
**SDI Regions**						
High SDI	54089.63(51454.43,56133.53)	48162.41(45410.91,51448.93)	11.74(11.17,12.18)	9.59(9.04,10.24)	-18.29(-22.22,-12.64)	-0.93(-1.15,-0.70)
High-middle SDI	82830.69(64720.17,93170.64)	74429.18(65515.59,85787.49)	14.68(11.47,16.51)	11.82(10.41,13.63)	-19.44(-33.02,2.49)	-1.13(-1.31,-0.95)
Middle SDI	84399.97(65233.09,95123.50)	99594.74(87511.54,114203.99)	9.27(7.16,10.45)	7.94(6.97,9.10)	-14.38(-29.04,13.17)	-0.64(-0.74,-0.54)
Low-middle SDI	42210.38(29424.64,48462.61)	64209.67(55704.86,77563.99)	7.66(5.34,8.79)	6.32(5.48,7.63)	-17.51(-33.98,25.84)	-1.07(-1.31,-0.84)
Low SDI	15981.78(13831.56,18664.35)	37336.14(31037.37,45508.59)	7.23(6.26,8.44)	6.88(5.72,8.39)	-4.79(-22.07,15.94)	-0.32(-0.41,-0.22)
**GBD Geographic Regions**						
East Asia	101919.77(67494.55,120901.32)	85364.44(67920.52,109266.39)	14.80(9.80,17.55)	12.40(9.86,15.87)	-16.20(-38.94,29.67)	-0.90(-1.10,-0.69)
Southeast Asia	12585.83(10728.30,14404.63)	22846.64(19112.15,27443.50)	5.32(4.53,6.09)	6.16(5.15,7.40)	15.83(-5.26,41.59)	0.32(0.16,0.48)
Oceania	177.58(104.25,240.94)	531.69(304.22,760.09)	5.56(3.26,7.54)	7.52(4.30,10.74)	35.22(2.88,84.83)	1.18(1.10,1.27)
Central Asia	3349.83(2961.98,3783.15)	4296.80(3700.46,4980.04)	10.05(8.88,11.34)	8.81(7.59,10.21)	-12.28(-26.61,6.09)	-1.17(-1.58,-0.76)
Central Europe	9751.79(9333.13,10201.75)	8533.20(7784.01,9363.50)	15.70(15.03,16.43)	16.20(14.77,17.77)	3.13(-5.63,12.89)	-0.56(-0.91,-0.21)
Eastern Europe	14763.10(13766.53,16375.29)	12130.31(10895.36,13472.41)	13.39(12.48,14.85)	12.61(11.32,14.00)	-5.83(-18.31,7.21)	-0.96(-1.55,-0.36)
High-income Asia Pacific	6728.90(6256.72,7214.19)	5136.28(4759.42,5623.42)	7.25(6.74,7.77)	6.57(6.08,7.19)	-9.42(-16.96,-1.24)	-0.46(-0.56,-0.36)
Australasia	966.95(882.89,1053.59)	1039.30(918.41,1170.93)	8.96(8.18,9.76)	7.20(6.36,8.11)	-19.68(-30.24,-6.76)	-0.69(-0.80,-0.57)
Western Europe	29726.38(28757.46,30865.48)	19559.21(18544.55,20720.03)	15.37(14.87,15.96)	10.38(9.84,10.99)	-32.49(-35.66,-28.98)	-1.25(-1.43,-1.07)
Southern Latin America	2838.06(2638.98,3059.14)	2603.48(2373.16,2870.64)	11.59(10.78,12.49)	7.51(6.84,8.28)	-35.23(-42.63,-27.31)	-1.50(-1.60,-1.40)
High-income North America	14613.49(13973.41,15389.63)	15400.48(14573.81,16354.03)	9.81(9.38,10.33)	9.13(8.64,9.70)	-6.88(-10.26,-3.53)	-0.49(-0.91,-0.06)
Caribbean	1353.25(1232.27,1486.69)	1893.06(1575.09,2257.71)	7.41(6.75,8.14)	7.91(6.58,9.43)	6.72(-9.57,25.30)	0.43(0.34,0.52)
Andean Latin America	959.22(842.22,1098.21)	1737.12(1403.30,2159.07)	5.15(4.52,5.89)	4.97(4.01,6.17)	-3.52(-25.00,20.65)	-0.32(-0.49,-0.15)
Central Latin America	4412.92(4281.42,4574.14)	7837.52(6977.45,8716.06)	5.41(5.25,5.60)	5.89(5.24,6.55)	8.90(-3.76,21.43)	0.23(0.06,0.40)
Tropical Latin America	5896.87(5624.42,6184.74)	9545.92(9121.40,10024.19)	7.51(7.16,7.88)	7.97(7.61,8.37)	6.07(0.46,12.06)	0.04(-0.16,0.23)
North Africa and Middle East	27233.41(16469.27,32419.08)	32296.58(26875.39,40691.06)	16.99(10.28,20.23)	9.66(8.04,12.17)	-43.15(-57.34,4.74)	-2.46(-2.91,-2.01)
South Asia	24398.31(20453.26,27978.58)	50331.86(42682.99,60576.65)	4.61(3.87,5.29)	5.00(4.24,6.02)	8.41(-11.69,35.80)	0.07(-0.00,0.15)
Central Sub-Saharan Africa	1831.66(1409.44,2285.12)	4954.46(3693.42,6470.39)	7.50(5.77,9.36)	7.60(5.66,9.92)	1.29(-27.35,41.03)	0.04(-0.06,0.15)
Eastern Sub-Saharan Africa	6963.11(5983.53,8192.72)	15628.66(12027.80,20543.07)	8.35(7.17,9.82)	7.46(5.74,9.81)	-10.59(-33.42,20.02)	-0.60(-0.73,-0.48)
Southern Sub-Saharan Africa	3977.39(3424.82,4413.23)	7739.82(6426.61,9294.28)	15.44(13.30,17.13)	17.93(14.89,21.53)	16.10(-5.84,41.94)	0.51(0.21,0.81)
Western Sub-Saharan Africa	5340.89(4305.44,6475.34)	14630.83(11560.23,18921.88)	6.24(5.03,7.56)	6.38(5.04,8.25)	2.27(-20.96,29.80)	0.08(0.02,0.14)

Table S2. Numbers of DALYs , DALYs ASR, EAPC DALYs ASR in 1990 and 2021 from Global Disease Burden 2021
