# Supplementary figures and images for: Unequal Progress in Early-Onset Bladder Cancer Control: Global Trends, Socioeconomic Disparities, and Policy Efficiency from 1990 to 2021
Source: Healthcare (Basel). 2026 Jan 12;14(2):193. doi: 10.3390/healthcare14020193 (PMC12840968; doi:10.3390/healthcare14020193)

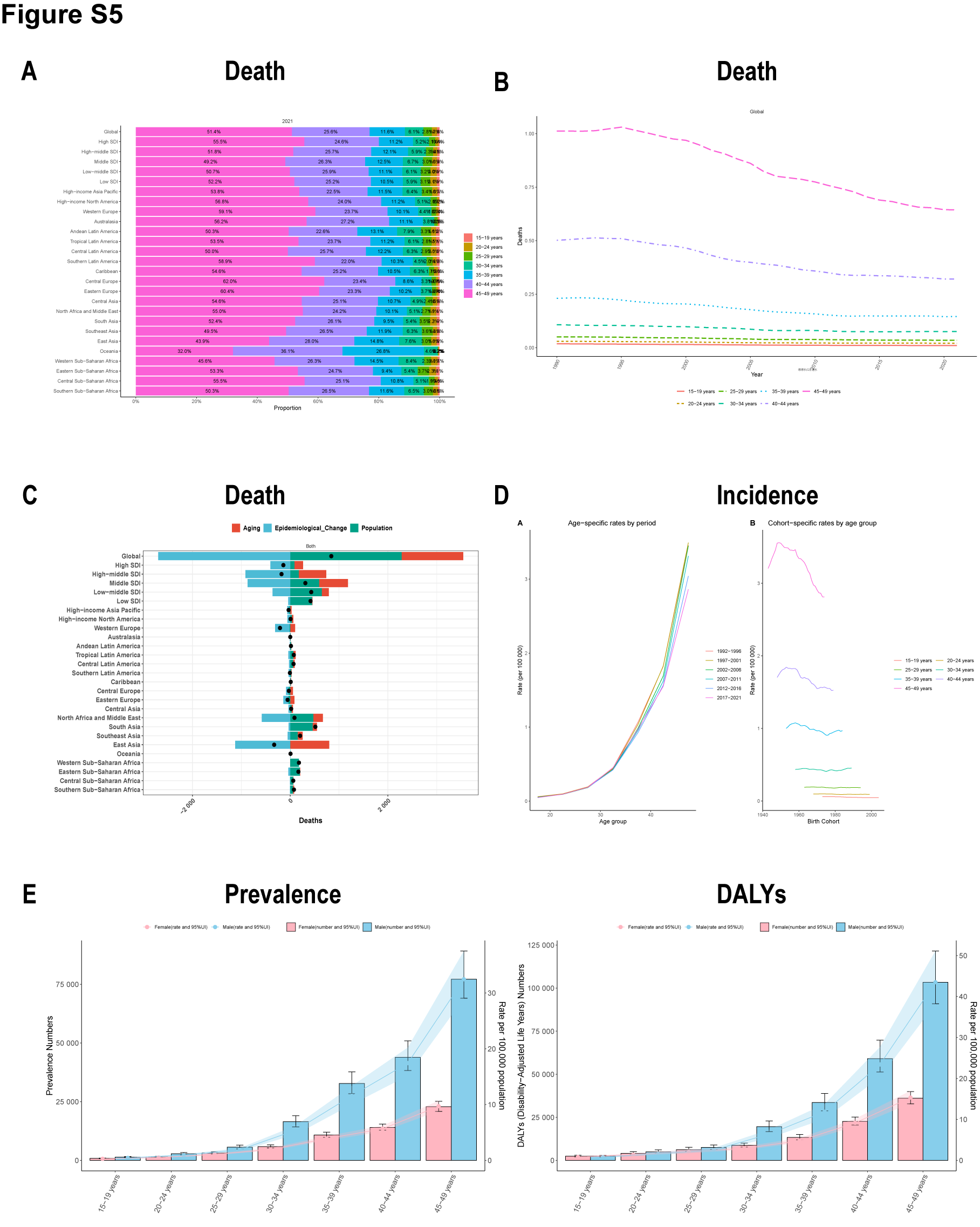

Supplement: Supplementary file 1 [file healthcare-14-00193-s001.zip › Figure S5.tif]

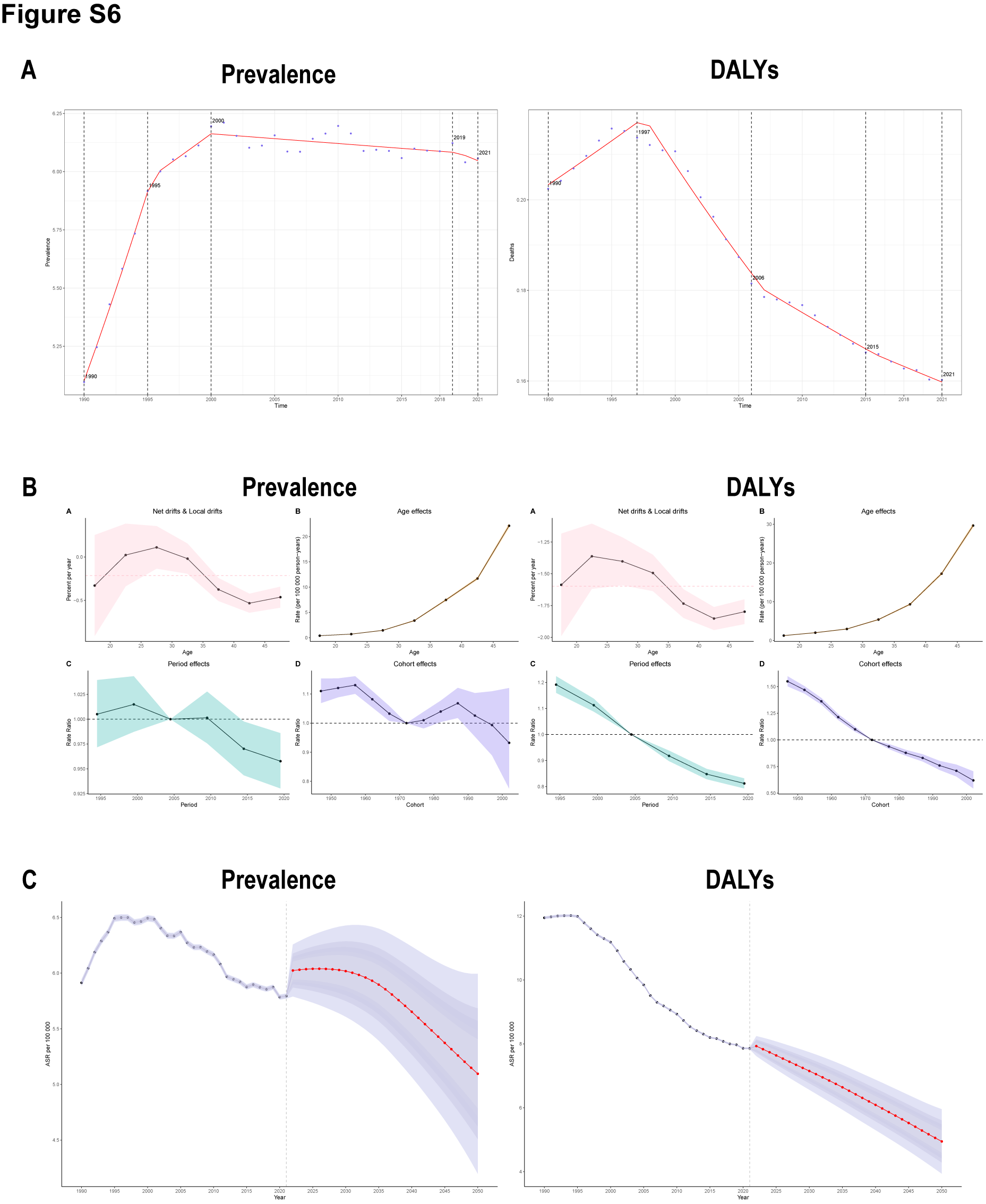

Supplement: Supplementary file 1 [file healthcare-14-00193-s001.zip › Figure S6.tif]

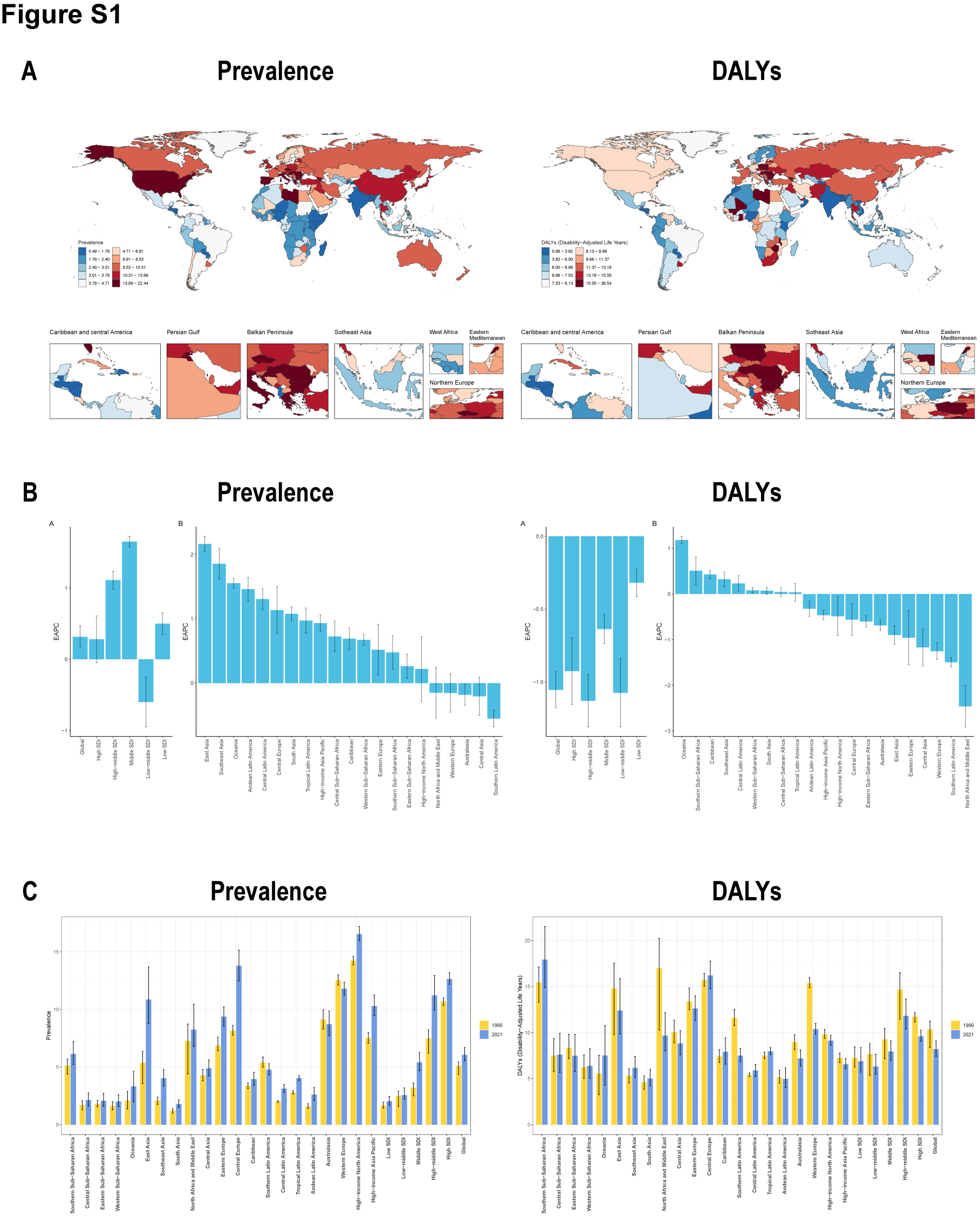

Supplement: Supplementary file 1 [file healthcare-14-00193-s001.zip › Figure S1.tif]

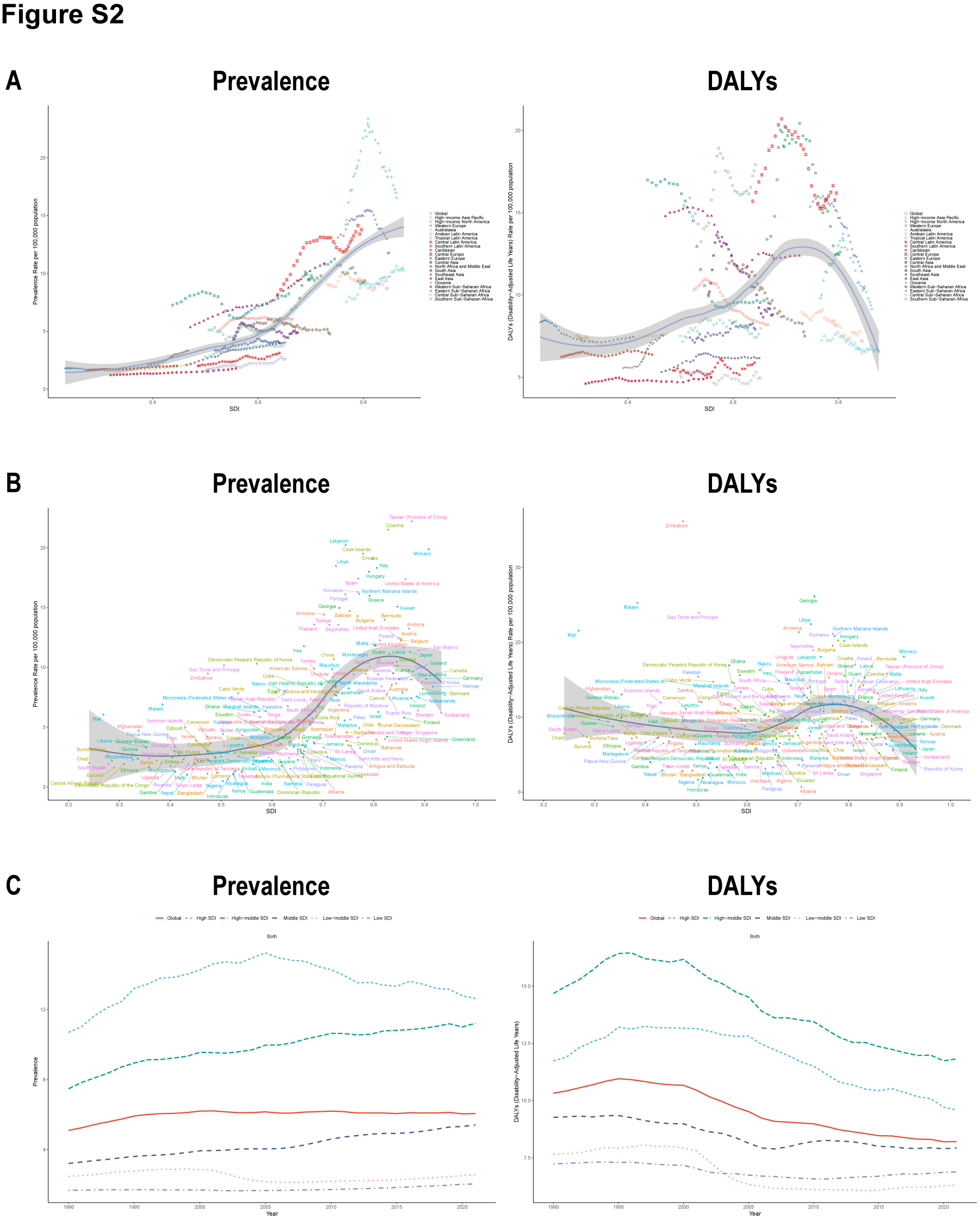

Supplement: Supplementary file 1 [file healthcare-14-00193-s001.zip › Figure S2.tif]

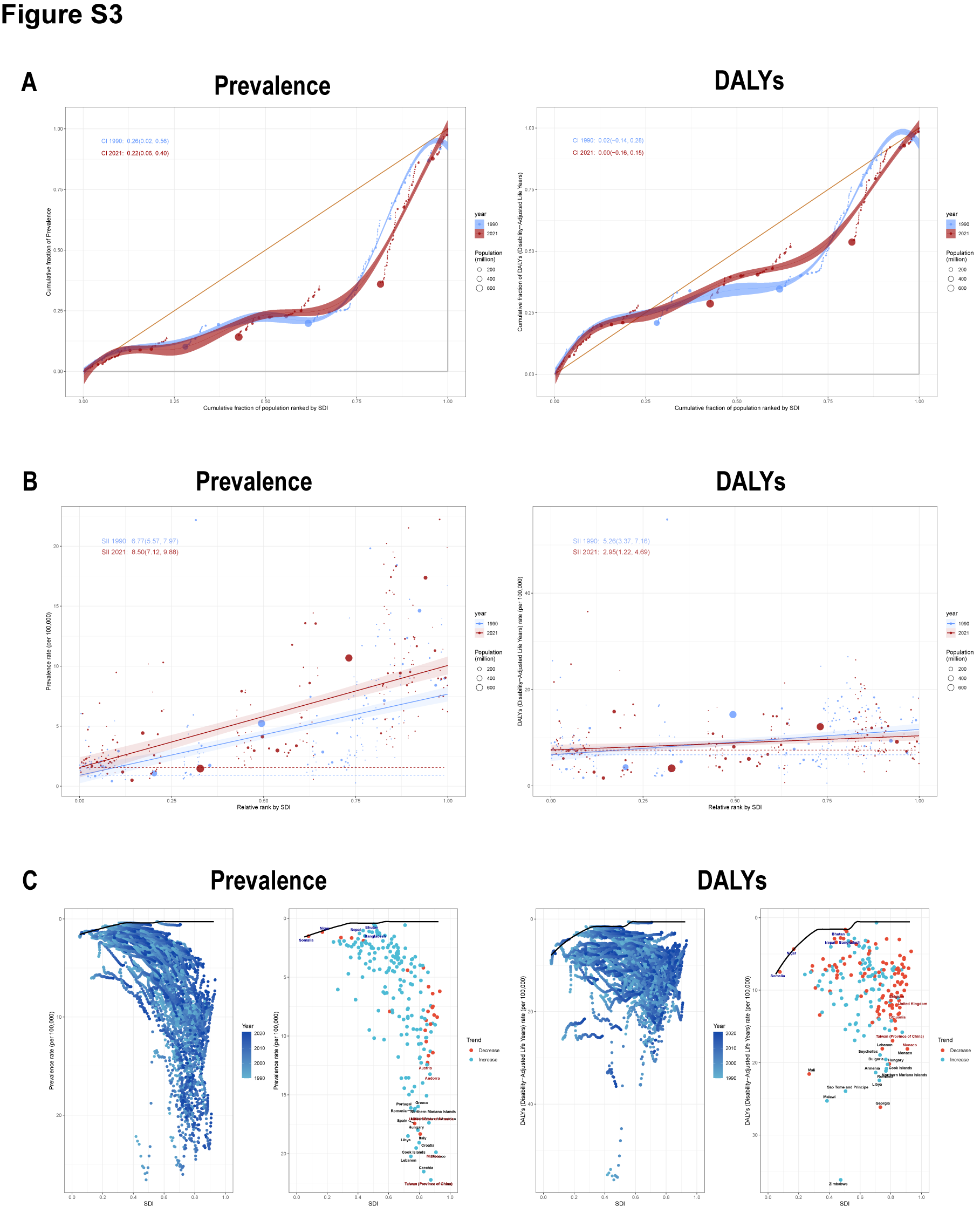

Supplement: Supplementary file 1 [file healthcare-14-00193-s001.zip › Figure S3.tif]

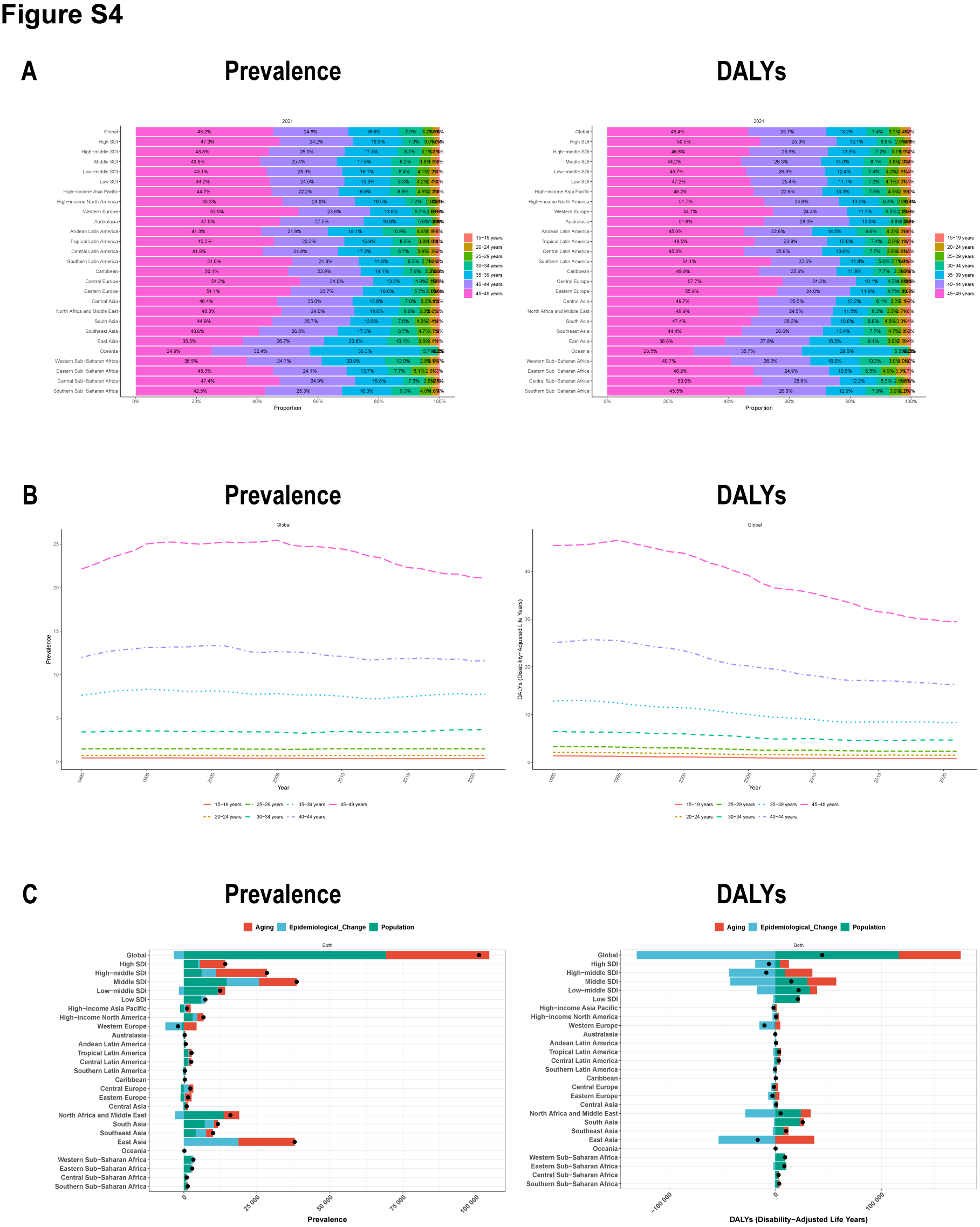

Supplement: Supplementary file 1 [file healthcare-14-00193-s001.zip › Figure S4.tif]
